# Mixture of Five Fermented Herbs (*Zhihuasi Tk*) Alters the Intestinal Microbiota and Promotes the Growth Performance in Piglets

**DOI:** 10.3389/fmicb.2021.725196

**Published:** 2021-10-26

**Authors:** Yong Li, Tiehu Sun, Yuxuan Hong, Tong Qiao, Yongsheng Wang, Wei Li, Shi Tang, Xin Yang, Jie Li, Xiaowen Li, Zutao Zhou, Yuncai Xiao

**Affiliations:** ^1^COFCO Feed Co., Ltd., Beijing, China; ^2^COFCO Nutrition and Health Research Institute, Beijing, China; ^3^College of Veterinary Medicine, Huazhong Agricultural University, Wuhan, China; ^4^State Key Laboratory of Agricultural Microbiology, Huazhong Agricultural University, Wuhan, China; ^5^Hubei Huada Real Science & Technology Co., Ltd., Wuhan, China

**Keywords:** weaned piglets, fermented Chinese herbal mixture, antioxidant, growth performance, intestinal microbiota

## Abstract

To explore the feasibility of using fermented Chinese herbal mixture *Zhihuasi Tk* (*Z. Tk*) supplementation to increase the swine production, the protective effect of dietary supplementation with *Z. Tk* on the intestinal oxidative stress model and the regulation of both growth performance and intestinal microbiota of weaned piglets were investigated *in vitro*. Our results showed that the addition of *Z. Tk* increased the cell viability, prevented the decrease of glutathione peroxidase, and significantly increased the total antioxidant capacity and reduced the damage caused by H_2_O_2_ to the tight junction proteins of the porcine small intestinal epithelial cell line (IPEC-J2). Furthermore, weaned piglets supplemented with either 2 kg/ton zinc oxide (ZnO) or 4 kg/ton of *Z. Tk* in the diet increased body weight as well as average daily feed intake and daily gain, while the feed conversion rate and diarrhea rate decreased within 0–35 days. Results of the taxonomic structure of the intestinal microbiota showed that, in 21 days after weaning, the Firmicutes/Bacteroidetes ratio in experimental group was increased, while the abundance of beneficial bacteria such, as *Lactobacillus*, was increased by *Z. Tk*, showing inhibitory effect on pathogenic bacteria such as members of Proteobacteria. In summary, dietary supplementation with *Z. Tk* maintained the intestinal microbiota in a favorable state for the host to effectively reduce the abnormal changes in the intestinal microbial structure and improved growth performance of weaned piglets. Therefore, *Z. Tk* may potentially function as a substitute for ZnO in feed additives for weaned piglets in modern husbandry.

## Introduction

In order to meet the production goals in the swine industry, piglets are generally weaned early prior to the establishment of a constant microbiota and a fully developed immune system (Odle et al., 1996). As one of the most traumatic stresses in a pig’s life, weaning can disrupt the gut microbial environment and increase susceptibility to post-weaning diarrhea caused by bacteria, ultimately leading to significant morbidity and mortality (Frydendahl, 2002; Campbell et al., 2013). To relieve the adverse effect of the anxiety on weaned piglets, effective strategies are needed to enhance gut health and improve the growth performance of weaned piglets while maintaining the standard of safety for consumers and farmer’s productivity (Xu et al., 2018; Chen et al., 2020).

Zinc is an essential trace element for animals. Due to its high efficiency and appropriate price, zinc is commonly used in the form of ZnO in high doses (2,000–3,000mg/kg diet) for weaned piglets as an alternative to antibiotics to prevent intestinal inflammation and increase body weight (Hu et al., 2012; Kociova et al., 2020). However, the large amount of zinc cannot be fully utilized by animals, causing severe environmental pollution (Kociova et al., 2020) and affect the intestinal microbiota by promoting the antimicrobial resistance (Bednorz et al., 2013; Rensing et al., 2018). Therefore, it is urgent to identify the safe alternatives of zinc to enhance the protection of both animals and environments (Liu et al., 2020).

Traditional Chinese medicine (TCM) commonly used in feed additives can not only effectively treat various types of medical disorders but also maintain general health and prevent disease in animals (Cao et al., 2015; Du et al., 2018; Li et al., 2019). Studies have shown that *Codonopsis pilosula*, as one of the traditional Chinese medicinal plants, has been used to effectively enhance immunity and improve microcirculation (Fu et al., 2018), while another popular medicinal herbal plant *Radix astragalus* has shown pharmaceutical effects on immunomodulation and anti-oxidation activation (Gong et al., 2018). It has been reported that the extract of *R. isatidis* showed the antioxidant and anti-inflammatory effects *in vitro* (Xiao et al., 2014). As the active ingredients of *Atractylodes macrocephala*, Atractylenolides contain the antioxidant and anti-inflammatory properties (Bailly, 2020). Studies have shown that *R. paeoniae alba* contains a large number of pharmacological properties including anti-inflammatory, antioxidation, improving immunity (Li et al., 2011; Su-Hong et al., 2015), and hepatoprotective effect (Zhao et al., 2020). It has been demonstrated that the mixture of herbal extracts contains an enhanced biological efficiency than a single extract because of the synergetic effects between the bioactive constituents of herbal mixture (Lan et al., 2017). Due to multiple pathogenic factors regulating many diseases, it is generally challenging to deal with these medical disorders. Furthermore, the functions of a single herbal medicine are often limited in treating these complicated diseases, incapable of efficiently addressing the multivariate conditions in patients. In the practice of traditional Chinese medicine, in order to achieve the enhanced therapeutic effect, several herbal medicines are commonly combined based on their medicinal properties to achieve the synergetic functions in treating the complicated diseases. To date, the explicit molecular mechanisms of herbal compatibility are still not fully elucidated. Studies have shown that the synergistic effects of herbal medicines are achieved by applying a pair of herbs with ingredients of similar therapeutic functions. For example, the active ingredients in one herbal medicine enhance the therapeutic effects of the ingredients in the other herbal medicine by regulating their absorption, distribution, metabolism, and excretion (Wang et al., 2012). Moreover, the inactive ingredients in the herbal medicines used individually become active used in combination (Wang et al., 2012). These results suggest that the therapeutic effects observed in our study are likely achieved by the combined application of these five herbal medicines.

The gut microbiota regulates physiological functions related to metabolism, biotransformation, and biosynthesis of biological ingredients (Krishnan et al., 2015; Kawai et al., 2018). Medical studies have demonstrated that microbial regulation may play an important role in the treatment of TCMs (Xu et al., 2015; Tong et al., 2018). For example, the gut microbiota may enhance the bioavailability and therapeutic effects of TCMs by regulating their transformation and absorption from the intestine into the blood, indicating that TCMs function depending on specific components of the gut microbiota (Zhang et al., 2020). Furthermore, TCMs may alter the taxonomic structure and metabolic production of the gut microbiota. For example, studies have shown that TCMs change the relative abundance of the gut microbiota at various taxonomic levels (Wei et al., 2018; Chen et al., 2018b). Therefore, it is highly speculated that the therapeutic efficiency of TCMs are, at least partially, attributed to the alteration in the gut microbiota.

A type of TCM called *Zhihuasi Tk* (*Z. Tk*) is a mixture of five well-known Chinese herbal medicines, including *Codonopsis pilosula* (Dangshen), Radix astragalus (Huangqi), R. isatidis (Banlangen), R. paeoniae alba (Baishao), and Atractylodes macrocephala (Baizhu), fermented first with probiotics and then dried and crushed. The purpose of microbial fermentation is usually to decompose or convert undesired substances into compatible materials, ultimately enhancing the product properties by increasing the amount of biologically effective compounds (Ugural and Akyol, 2020). Furthermore, the fermentation is revealed to strengthen the antioxidant effects of some plants (Kusznierewicz et al., 2008; Ugural and Akyol, 2020). For example, fermentation increases the content of phenolic in some plant products (Đorđević et al., 2010), and it is reported that the polyphenols are positively correlated with the antioxidant activities in medicinal herbs (Shan et al., 2005). Recently, the Chinese herbs have attracted growing attention as feed additives in animal production. However, studies on the effects of Chinese herbs on weaned piglets are sparse. The main purpose of this study was to explore the protective effect of *Z. Tk* on the oxidative damage of the epithelial cell barrier and to investigate the potential effect of this feed additive on improving the intestinal microbiota and the growth performance of weaned piglets.

## Materials and Methods

### Preparation of *Zhihuasi Tk* Extracts

The *Zhihuasi Tk* (*Z. Tk*) is a mixed combination of five commonly used TCMs with different proportions including *Codonopsis pilosula* (20%), *Radix astragalus* (30%), *R. isatidis* (15%), *R. paeoniae alba* (25%), and *Atractylodes macrocephalae* (10%). The mixture was infiltrated in water to 50% moisture, heated to 80°C for 1 h, and then cooled to 37°C. Three species of probiotics (*Bacillus subtilis*, *Enterococcus faecalis*, and *Saccharomyces cerevisiae*) were simultaneously added into the processed Chinese herbal mixture, which was fermented for 72 h, then dried, and crushed into powder. We selected the fermentation based on multi-strain probiotics, instead of a single-strain probiotics, due to the more comprehensive effects of multiple bacteria and enzymes, providing higher fermentation efficiency and richer fermentation products, in particular with the medicinal fungi and probiotics. The three species of probiotics (i.e., *Bacillus subtilis*, *Enterococcus faecalis*, and *Saccharomyces cerevisiae*) used in our study are commonly applied in the fermentation of Chinese herbal medicines with sound probiotic properties (Li et al., 2020).

To investigate the effects of *Z. Tk* on the porcine small intestinal epithelial cell line (IPEC-J2), a total of 10 g of *Z. Tk* powder were submerged into 200 μl 100°C sterilized ultrapure water and incubated for 30 min in a water bath of 100°C. The large debris in the supernatant was filtered using a 300-mesh filter and then centrifuged for 30 min at 100 *g* to remove the small debris. Then, the supernatant was filtered with a 100-mesh cell sieve and used for cell culture.

### IPEC-J2 Cell Culture

The IPEC-J2 cell lines were provided by the College of Veterinary Medicine, Huazhong Agricultural University. These cells were maintained in the Dulbecco’s Modified Eagle Medium/Nutrient Mixture F-12, containing 10% fetal bovine serum and 1% penicillin–streptomycin (Gibco BRL Co., Ltd., United States), and cultured in an incubator with constant temperature (37°C) and atmosphere of 5% CO_2_. The medium was replaced every 3 days. Then, the cells were subcultured with 0.05% trypsin (Gibco BRL Co., Ltd., United States). The monolayers of cells were collected after 3 days of incubation for further experiments.

### IPEC-J2 Cell Proliferation

The IPEC-J2 cells grown at logarithmic phase were treated with 0.1% trypsin to prepare a single cell suspension and then seeded in a 96-well cell culture plate. The number of seeding was 1 × 10^4^ cells/well and cultivated for 24 h in an incubator (37°C) with constant concentration of CO_2_ (5%). Then, the medium was discarded, the wells were washed with sterile PBS, added with medium containing 0, 1, 5, 10, 50, 100, 200, 500, 800, and 1,000 μM/L H_2_O_2_, respectively, and cultured for 24 h (six replicate wells per group). For each well, the culture medium was discarded, added with 100 μl of medium containing 10% Cell Counting Kit-8 (Biosharp, China), and incubated for 2 h at 37°C. The OD_450_ value was measured with a microplate reader. The concentration of H_2_O_2_ applied in the subsequent experiments was determined based on its reduction of cell viability by 50% with the cells treated with H_2_O_2_ for 24 h to establish a cellular oxidative stress model. For the treatment of *Z. Tk*, the cells were treated with the medium containing 1 × 10^−2^, 5 × 10^−3^, 1 × 10^−3^, 5 × 10^−4^, 1 × 10^−4^, and 5 × 10^−5^ dilutions of *Z. Tk* extracts for 3 h, then added 100 μl of medium containing 10% Cell Counting Kit-8 (Biosharp, China), incubated at 37°C for 2 h, and the OD_450_ values were measured with a microplate reader.

### Cellular Antioxidant Assay

Based on different treatments, the IPEC-J2 cells were separated into four groups, including the control group, the H_2_O_2_ oxidative stress group (treated with H_2_O_2_ for 24 h), the *Z. Tk* group (treated with *Z. Tk* for 3 h), and the *Z. Tk* + H_2_O_2_ group (treated with *Z. Tk* for 3 h and then H_2_O_2_ for 24 h) with each group containing three replicate wells and cultivated for 24 h. The cell culture fluid was collected to detect the total antioxidant capacity (T-AOC) and measure the content of glutathione peroxidase (GSH-Px) using the total antioxidant capacity test kit (Shanghai Biyuntian Biotechnology Company, Shanghai, China) and the glutathione peroxidase test kit (Shanghai Biyuntian Biotechnology Company, Shanghai, China) following the manufacturer’s instructions, respectively. The total RNA was extracted using the Total RNA Kit (R6812, U.S. Omega Bio-Tek Company) and used to synthesize cDNA using the FastKing reverse transcription kit (KR118-02, Beijing Tiangen Biological Co., Ltd., Beijing, China). The expressions of three genes (*ZO-1*, *occludin*, and *claudin*) encoding the tight junction proteins in IPEC-2 cells were determined using qPCR method with the primers shown in Table 1.

**Table 1:** Primer sequences for genes amplified in this study. F, forward primer; R, reverse primer.

Gene	Primer sequence (5ʹ-3ʹ)	Amplified fragment (bp)
*ß-Actin*	F: TGCGGGACATCAAGGAGAAG R: AGTTGAAGGTGGTCTCGTGG	92
*claudin*	F: GGCCCTTACCTTTCGCCTGA R: GCCTCAGGGCTTGGTGTTCT	99
*occludin*	F: ATCAACAAAGGCAACTCT R: GCAGCAGCCATGTACTCT	74
*ZO-1*	F: GCCTCAGGGCTTGGTGTTCT R: GGCCCTTACCTTTCGCCTGA	87

### Laboratory Animals

Our study was performed in accordance with the Guide for the Care and Use of Laboratory Animals Monitoring Committee of Hubei Province, China with the protocols approved by the Committee on the Ethics of Animal Experiments of the College of Veterinary Medicine, Huazhong Agricultural University (No. HZAUSW-2020-0001).

A total of 135 healthy Duroc × Landrace × Yorkshine hybrid (Du Changda) weaned boars (weaned at 23 days of age) were selected and evenly divided into three groups based on their body weight (BW) with each group of nine replicates and five piglets per replicate. The negative control (NC) group was fed with basal diet, the positive control (PC) group was fed with basal diet plus 2 kg/ton zinc oxide (ZnO), and the experimental group was fed with the basal diet plus 4 kg/ton of *Z. Tk*. The pharmacological dose of 2 kg/ton to feed the weaned piglets as a positive control was based on a previous study (Wang et al., 2019). Results of our preliminary experiments using the concentrations of 1 kg/ton, 2 kg/ton, and 4 kg/ton showed that 4 kg/ton generated the highest economic benefit. Therefore, we used 4 kg/ton as the concentration of *Z. Tk* in our experiments. No animals were fed with antibiotics. The nutrient levels of crude protein, calcium, and total phosphorus in the diets of each group were consistent and in accordance with both the NRC (2012) and the Chinese Pig Feeding Standard (2004). The detailed feed formula and the nutritional levels were shown in Supplementary Tables S1 and S2, respectively.

The experiments were carried out at the experimental base of the Feed Research Institute of the Chinese Academy of Agricultural Sciences for a total of 35 days. A fully enclosed pig house was adopted with the temperature (24~26°C), humidity, ventilation intensity, and the carbon dioxide and ammonia concentrations in the house automatically controlled. The piglets were raised in pens each of 1.5 m × 1.5 m in size equipped with slatted plastic spray floor, adjustable stainless steel troughs, and nipple drinkers. Animals were fed with pellets and were free to eat and drink.

### Growth Performance

On days 0, 7, 21, and 35, the weaned piglets and feed were weighed to calculate the growth indices, including average daily gain (ADG), average daily feed intake (ADFI), and feed conversion rate (FCR) as follows: ADG = (end weight − initial weight)/days of experiment, ADFI = (feed amount provided during the test period − the remaining amount of feed during the test period)/days of experiment, and FCR = average daily feed intake/average daily gain of weight.

For the first 3 weeks, the condition of diarrhea of piglets was recorded twice a day to calculate the diarrhea rate based on the diarrhea score of each group of piglets. Specifically, 0 point was scored based on the cylindrical shaped soft stools, mild to moderate diarrhea (showing irregular loose stools with high water content) was scored as 1 point, and severe diarrhea characterized as having liquid, irregular, and watery loose stools was scored as 2 points. The diarrhea rate was calculated as the (number of piglets with diarrhea)/(number of piglets tested × total days) × 100%.

### Sample Collection, DNA Extraction, and Sequencing

On days 21 and 35, the feces of the piglets with the same ear size were collected from nin replicates of the three groups by rectal massage. The samples were immediately divided into the cryopreservation tubes and kept in liquid nitrogen tank (−196°C) for storage.

The total genomic DNA from fecal samples of the weaned piglets was extracted based on CTAB method (Ahrens, 1992), examined by 1% agarose gel electrophoresis, and diluted to 1 ng/μl with sterile water. PCR amplification of the V3–V4 region of the bacterial 16S rRNA gene was performed with the forward primer 341F (5ʹ-CCTAYGGGRBGCASCAG-3ʹ) and the reverse primer 806R (5ʹ-GGACTACHVGGGTWTCTAAT-3ʹ) with barcodes. PCR reactions contained 15 μl of Phusion® High-Fidelity PCR Master Mix (New England Biolabs), ~10 ng template DNA, and 2 μM of both forward and reverse primers. PCR procedures were as follows: initial denaturation at 98°C for 1 min, followed by 30 cycles of “denaturation at 98°C for 10 s, annealing at 50°C for 30 s, and elongation at 72°C for 30 s” with the final extension of 5 min at 72°C. The PCR products mixed with the same volume of 1X loading buffer (containing SYBR green) were detected using electrophoresis on 2% agarose gel. Then, the mixture of PCR products of equidensity ratios was purified with Qiagen Gel Extraction Kit (Qiagen, Germany).

Libraries for high-throughput sequencing were constructed using the TruSeq® DNA PCR-Free Sample Preparation Kit (Illumina, United States) following manufacturer’s protocols with index codes added. The quality of the sequencing libraries was evaluated by the Qubit@ 2.0 Fluorometer (Thermo Scientific) and Agilent Bioanalyzer 2,100 system (Agilent Technologies, United States). Samples with PCR amplified fragment size and PCR product volume not meeting the requirements for library construction were eliminated; samples with QC30 > 90% were selected for further analysis. The library was sequenced on an Illumina next-generation sequencing platform NovaSeq to generate the 250 bp paired-end reads. Furthermore, to avoid the inclusion of inappropriate genomic data to interfere the reliability of subsequent analyses, the results of the relative abundance and beta diversity analysis were used to eliminate samples showing significant differences within the group.

### Sequencing Data Analysis

Paired-end reads were aligned to samples based on their unique barcodes with the barcode and primer sequences removed. The reads were merged using FLASH V1.2.7 (Magoč and Salzberg, 2011). The high-quality clean reads were obtained by filtering the raw reads using the QIIME V1.9.1 with filtering conditions set as previously reported (Caporaso et al., 2010). The final set of effective reads was obtained by comparing the clean reads with the reference database SILVA using the UCHIME algorithm (Edgar et al., 2011) to remove the chimera sequences (Haas et al., 2011).

Sequences with ≥ 97% similarity were assigned to the same Operational Taxonomic Units (OTUs) using Uparse v7.0.1001 (Edgar, 2013). Further annotation was performed on the representative sequence of each OTU screened using the SSUrRNA database (Wang et al., 2007) of SILVA (Quast et al., 2013) using the Mothur algorithm with the threshold set at 0.8–1.0. Taxonomic rank and the microbial composition of each sample at seven ranks of classification (i.e., kingdom, phylum, class, order, family, genus, and species) were obtained to evaluate the difference between groups using T-test. Multiple sequence alignment was performed based on the MUSCLE Version 3.8.31 (Edgar, 2004) to investigate the phylogenetic relationship among OTUs and the variations of the abundant taxa in different samples and groups. The normalization of the abundance of OTUs was performed prior to the subsequent analyses of alpha diversity and beta diversity of the gut microbiota.

The complexity of species diversity was evaluated by generating alpha diversity indices, including the observed-species, the Chao1 estimator, and the Shannon index using QIIME Version 1.7.0 with the results (i.e., the dilution curve, the species accumulation curve, and the rank abundance curve) displayed with R software Version 2.15.3, which was also used to draw the dilution curve, the rank abundance curve, and the species accumulation curve. The Wilcox test in R software was used to analyze the difference of the alpha diversity indices between groups.

Beta diversity analyses on both weighted and unweighted UniFrac were conducted to evaluate the differences of samples in species complexity using QIIME Version 1.9.1. The principal coordinate analysis (PCoA) was performed to evaluate the principal coordinates and visualize the multidimensional data. A distance matrix of weighted or unweighted UniFrac among samples was generated to establish a new set of orthogonal coordinates, with the maximum variation factor demonstrated by the first principal coordinate, the second maximum variation factor by the second principal coordinate, and so on. The results of the PCoA analysis were displayed by WGCNA, stat, and ggplot2 packages in R software Version 2.15.3. The unweighted pair-group method with arithmetic means (UPGMA) analysis was performed based on the average linkage as a type of hierarchical clustering method to explain the distance matrix by QIIME Version 1.9.1.

### Statistical Analyses

Statistical analyses were performed using SPSS for Windows, version 22 (SPSS, Inc., Chicago, IL, United States). Graphs were generated using GraphPad Prism 5 software (GraphPad, Inc., California, United States). The data were presented as the mean ± standard error of the mean (SEM) with the significance levels for all analyses set as *p* < 0.05 (*) and *p* < 0.01 (**).

## Results

### Effects of H_2_O_2_ and *Z. Tk* on Cell Viability of IPEC-J2

**Figure 1:**
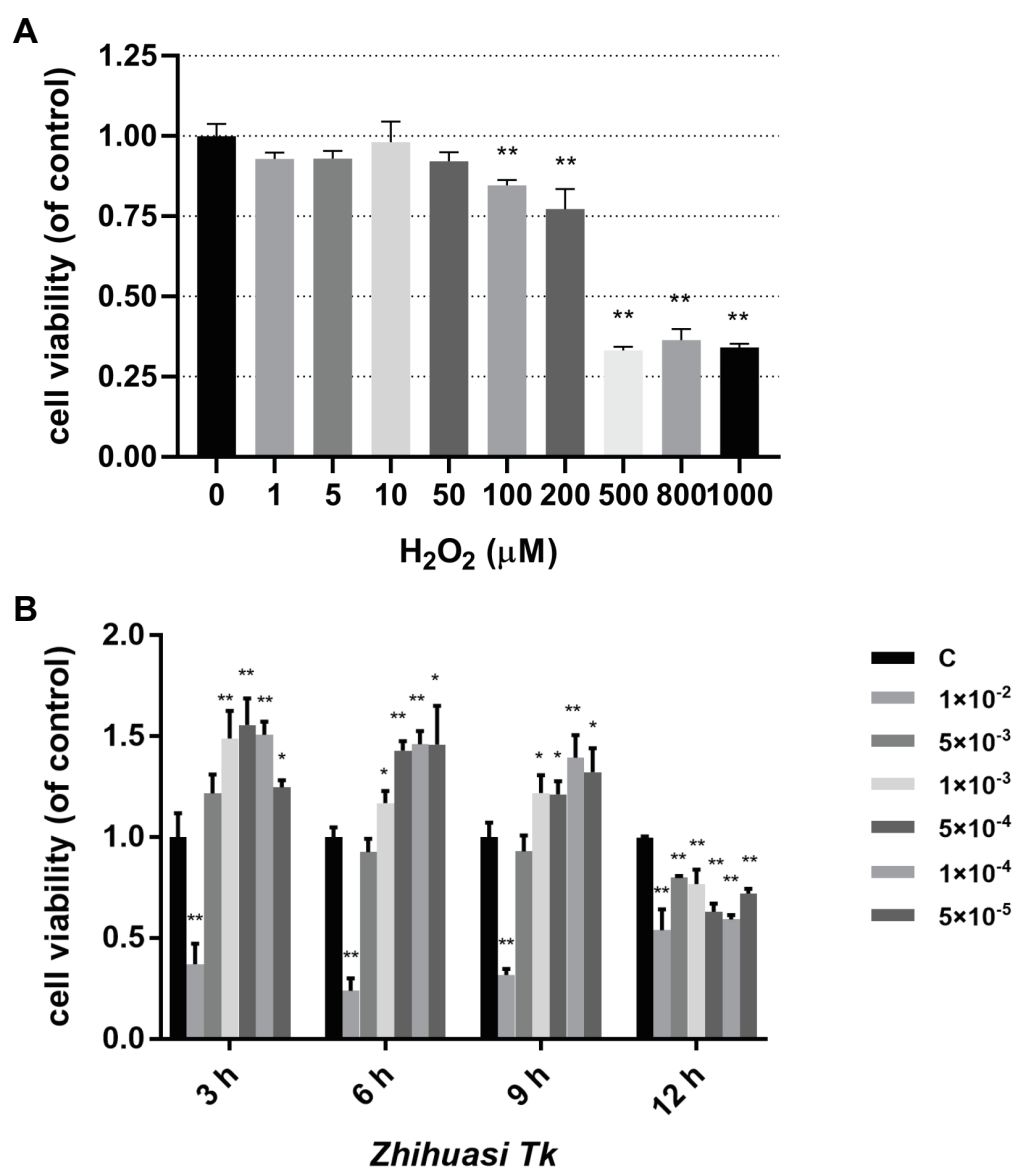
Effects of H_2_O_2_ **(A)** and *Z. Tk* **(B)** of different concentrations on the viability of IPEC-J2 cells treated with different times. Symbols “*” and “**” indicate the significant differences compared with the control group set at the values of *p* of 0.05 and 0.01, respectively.

The oxidative stress model of IPEC-J2 cells was established by the induction of H_2_O_2_. Results showed that the cell viability was significantly reduced under the treatment of 100 μM H_2_O_2_, while the IPEC-J2 cell viability was reduced to below 50% treated by H_2_O_2_ of 500–1,000 μM for 24 h in comparison to the control group (Figure 1A). Therefore, the concentration of 500 μM was chosen as the optimal content of H_2_O_2_ for the following experiments.

Results of the effect of *Z. Tk* on the viability of IPEC-J2 cells showed that both the high concentration of *Z. Tk* extract (i.e., 1 × 10^−2^) and longer incubation time inhibited cell proliferation (Figure 1B), while low concentrations of *Z. Tk* extract (e.g., 5 × 10^−4^ and 1 × 10^−4^) stimulated cells and significantly increased cell viability. Specifically, the cell viability was significantly improved by the stimulation of 3 h of all three dilutions of *Z. Tk* (i.e., 1 × 10^−3^, 5 × 10^−4^, and 1 × 10^−4^), with the dilution of 5 × 10^−4^ achieving the highest cell viability. Therefore, the dilution of 5 × 10^−4^ of *Z. Tk* and stimulation time for 3 h were selected for subsequent experiments.

### Antioxidant Effect of *Z. Tk*

**Figure 2:**
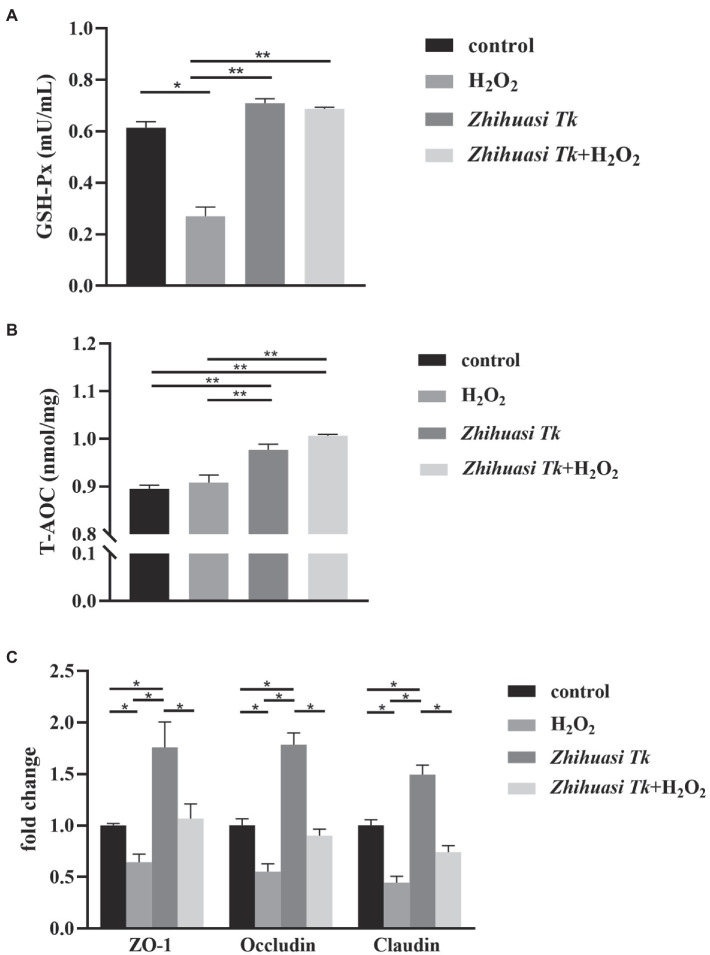
Protective effects of *Z. Tk* on IPEC-J2 cells under oxidative stress as indicated by the content of GSH-Px **(A)**, the T-AOC **(B)** in the supernatant of IPEC-J2 cells, and the expression of genes (i.e., *ZO-1*, *occluding*, and *claudin*) encoding the tight junction proteins in IPEC-J2 cells **(C)**. Symbols “*” and “**” indicate the significant differences set at the values of *p* of 0.05 and 0.01, respectively.

Results of the protective effects of *Z. Tk* on the IPEC-J2 cells under oxidative stress showed that after H_2_O_2_ stimulation, the content of GSH-Px in the supernatant of IPEC-J2 cells was decreased significantly in comparison to that of the control group, while the treatment of both *Z. Tk* and H_2_O_2_ significantly reduced the detrimental effect of H_2_O_2_, indicating that *Z. Tk* could protect the epithelium of the small intestine to prevent the decrease of GSH-Px (Figure 2A). Furthermore, the *Z. Tk* significantly improved the T-AOC of IPEC-J2 cells (Figure 2B). The expressions of three genes (*ZO-1*, *occludin*, and *claudin*) encoding the tight junction proteins were significantly reduced in IPEC-J2 cells treated with H_2_O_2_ in comparison to the control group, while *Z. Tk* significantly increased the expression of these genes, alleviating the detrimental effects of H_2_O_2_ on the tight junction proteins (Figure 2C). It was noted that the herbal extracts and H_2_O_2_ were not mixed throughout the entire process of treatment “*Z. Tk* + H_2_O_2_” group.

### Growth Performance of Piglets

The results of growth performance in the piglets are shown in Table 2. The initial weights of the weaned piglets remained the same among the three groups of animals. On days 21 and 35, the BW of the experimental group was significantly higher than that of the NC group (*p* < 0.05). On day 35, the BW increased on average by 17.31 and 13.77% in the experimental and PC groups, respectively, compared with the NC group, although the difference was not statistically significant in PC group. In 1–7, 8–21, and 1–35 days, the experimental group showed significantly higher ADG than that of the NC group (*p* < 0.05). In 35 days, the ADG of both the PC and experimental groups increased by 20.53 and 25.65%, respectively, in comparison to the NC group. Compared with the NC group, both the experimental and the PC groups showed increased trend of the ADFI and decreased trend of diarrhea rate in 1–35 days although the difference was not statistically significant. Both the experimental group (*p* < 0.05) and PC group (*p* < 0.01) showed significantly decreased FCR at 1–35 days, compared to the NC group.

**Table 2:** Growth performance in three groups of weaned piglets at different times.

	Negative control group	Positive control group	Test group
**BW (kg)**			
1 d	7.43 ± 0.45	7.46 ± 0.37	7.46 ± 0.37
7 d	9.27 ± 0.50	10.12 ± 0.52	10.29 ± 0.56
21 d	14.23 ± 0.85^a^	16.32 ± 0.77^ab^	16.89 ± 0.72^b^
35 d	21.14 ± 1.14^a^	24.05 ± 0.80^ab^	24.80 ± 1.12^b^
**ADG (g/d)**			
1–7 d	262.90 ± 19.92^A^	379.21 ± 25.89^B^	404.65 ± 28.69^B^
8–21 d	354.31 ± 27.30^a^	438.49 ± 23.44^b^	462.91 ± 26.66^b^
22–35 d	493.53 ± 37.61	552.23 ± 17.83	565.18 ± 31.71
1–35 d	391.71 ± 22.69^a^	472.13 ± 14.65^b^	492.17 ± 24.01^b^
**ADFI (g/d)**			
1–7 d	427.73 ± 20.31	478.21 ± 22.97	465.48 ± 25.29
8–21 d	527.42 ± 37.23	596.90 ± 30.87	614.75 ± 36.64
22–35 d	810.66 ± 44.08	883.56 ± 30.00	953.38 ± 65.27
1–35 d	620.78 ± 32.55	687.83 ± 27.65	720.35 ± 44.60
**FCR**			
1–7 d	1.70 ± 0.15^Aa^	1.29 ± 0.07^ABb^	1.16 ± 0.04^Bab^
8–21 d	1.51 ± 0.07	1.36 ± 0.03	1.34 ± 0.06
22–35 d	1.65 ± 0.05	1.61 ± 0.05	1.69 ± 0.06
1–35 d	1.59 ± 0.04^Aa^	1.45 ± 0.02^Bab^	1.46 ± 0.03^ABb^
**Diarrhea rate (%)**			
1–7 d	11.38 ± 4.91	8.16 ± 3.83	6.786 ± 2.22
8–14 d	11.16 ± 5.13	7.41 ± 3.76	6.65 ± 1.72
15–21 d	10.63 ± 3.43	4.11 ± 2.69	4.64 ± 2.47
1–21 d	6.93 ± 2.45	4.29 ± 2.02	3.96 ± 1.28

*BW, body weight; ADG, average daily gain; ADFI, average daily feed intake; FCR, feed conversion rate. Superscript letters a and b and superscript letters A and B in the same row indicate significant differences at *p* < 0.05 and p < 0.01, respectively*.

### Effect of *Z. Tk* on the Diversity of the Intestinal Microbiota of Weaned Piglets

To further explore the effects of *Z. Tk* on the intestinal microbiota of weaned piglets, the V3-V4 regions of the 16S rRNA gene were sequenced based on the genomic DNA extracted from feces of the weaned piglets. A total of 2,991,714 clean reads were obtained after screening and splicing of the raw reads with an average of 55,402 ± 835 reads per sample and an average length of 414 bp per read (Supplementary Table S3).

**Figure 3:**
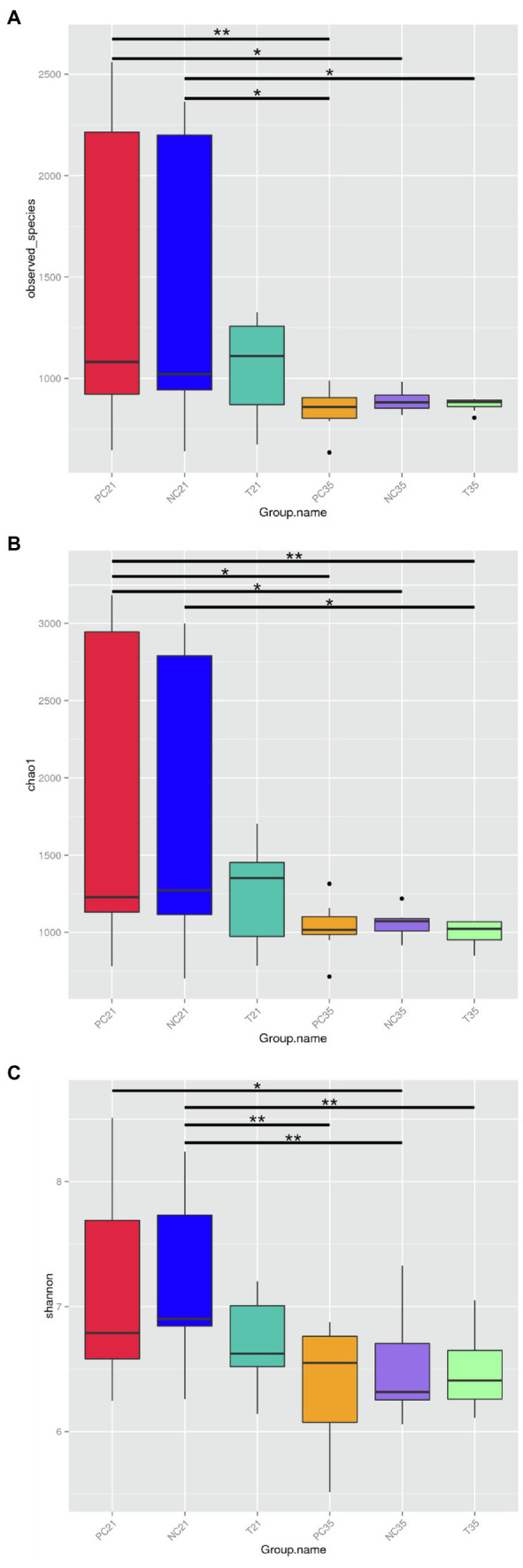
Effects of *Z. Tk* and ZnO on the intestinal microbiota diversity in weaned piglets in 21 and 35 days based on alpha diversity parameters of observed species **(A)**, Chao1 index **(B)**, and the Shannon index **(C)**. Symbols “*” and “**” indicate significant differences at *p* < 0.05 and *p* < 0.01, respectively. Each of the nine samples is selected from each of the three groups of piglets, i.e., the positive control (PC), the negative control (NC), and the test groups, on days 21 and 35, respectively.

The representative sequences of OTUs were obtained based on the clustered clean reads with 97% similarity. The rarefaction curve of each sample was generally flat, suggesting that the depth of sequencing was sufficient to cover all species in the sample (Supplementary Figure S1). Alpha diversity analyses were conducted based on the OTUs to determine three alpha diversity parameters, including the observed-species to show the observed OTUs (Figure 3A), the Chao1 index to estimate the community richness (Figure 3B), and the Shannon’s diversity index to evaluate the community diversity indices (Figure 3C). Result revealed no significant difference in Shannon diversity among the NC, PC, and experimental groups on either day 21 or day 35 (*p* > 0.05), while the NC group on day 35 showed significantly lower Shannon’s diversity index than that on day 21 (*p* < 0.01). For the community richness comparison, no significant difference was revealed in the number of observed species (OTUs) and estimated community richness (Chao1 index) among the NC, PC, and experimental groups on either day 21 or 35 (*p* > 0.05), while the observed-species (*p* < 0.01) and the Chao1 indices (*p* < 0.05) in the PC group changed significantly between days 21 and 35. Compared with the 21-day-old piglets, the community richness and the observed-species of PC group decreased significantly on day 35.

**Figure 4:**
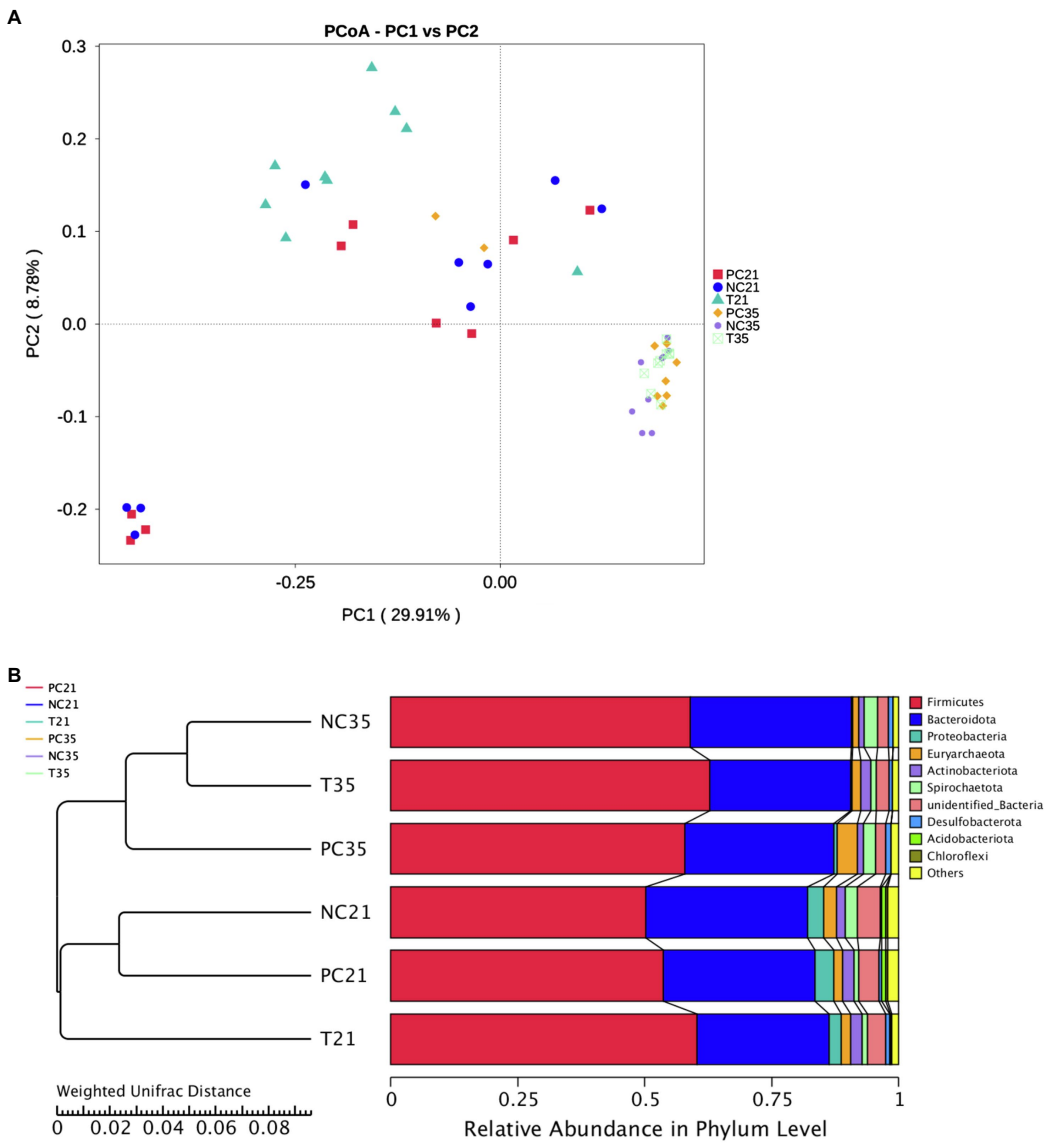
Effects of *Z. Tk* and ZnO on the intestinal microbial diversity in weaned piglets in 21 and 35 days based on beta diversity analyses of principal coordinate analysis **(A)** and UPGMA clustering analysis **(B)** based on weighted UniFrac. Each of the nine samples is selected from each of the three groups of piglets, i.e., the positive control (PC), the negative control (NC), and the test groups, on days 21 and 35, respectively.

The effects of *Z. Tk* on the intestinal microflora profile of weaned piglets were further investigated using the PCoA based on weighted UniFrac of OTUs (Figure 4A). Results showed that in 21 days, the experimental group was relatively separated from the NC and PC groups on the PCoA scatter plot. In 35 days, no clear separation was observed among the three groups of animals, indicating the similarities among the three groups of gut microbiota. The UPGMA clustering tree based on weighted UniFrac showed that the six groups of fecal microbial communities were revealed on two branches corresponding to the ages (i.e., 21 and 35 days) of the piglets (Figure 4B). In 21 days, the microbial community in the feces of the NC group was closely related to that of the PC group. This result was consistent with that revealed by the PCoA. In 35 days, the NC and experimental groups were clustered into the same branch. The close relationship between the PC and NC groups indicated that *Z. Tk* significantly altered the composition of the intestinal microbiota of weaned piglets in their early weaning phases.

### Effects of *Z. Tk* on the Taxonomic Composition of the Intestinal Microbiota in Weaned Piglets

**Figure 5:**
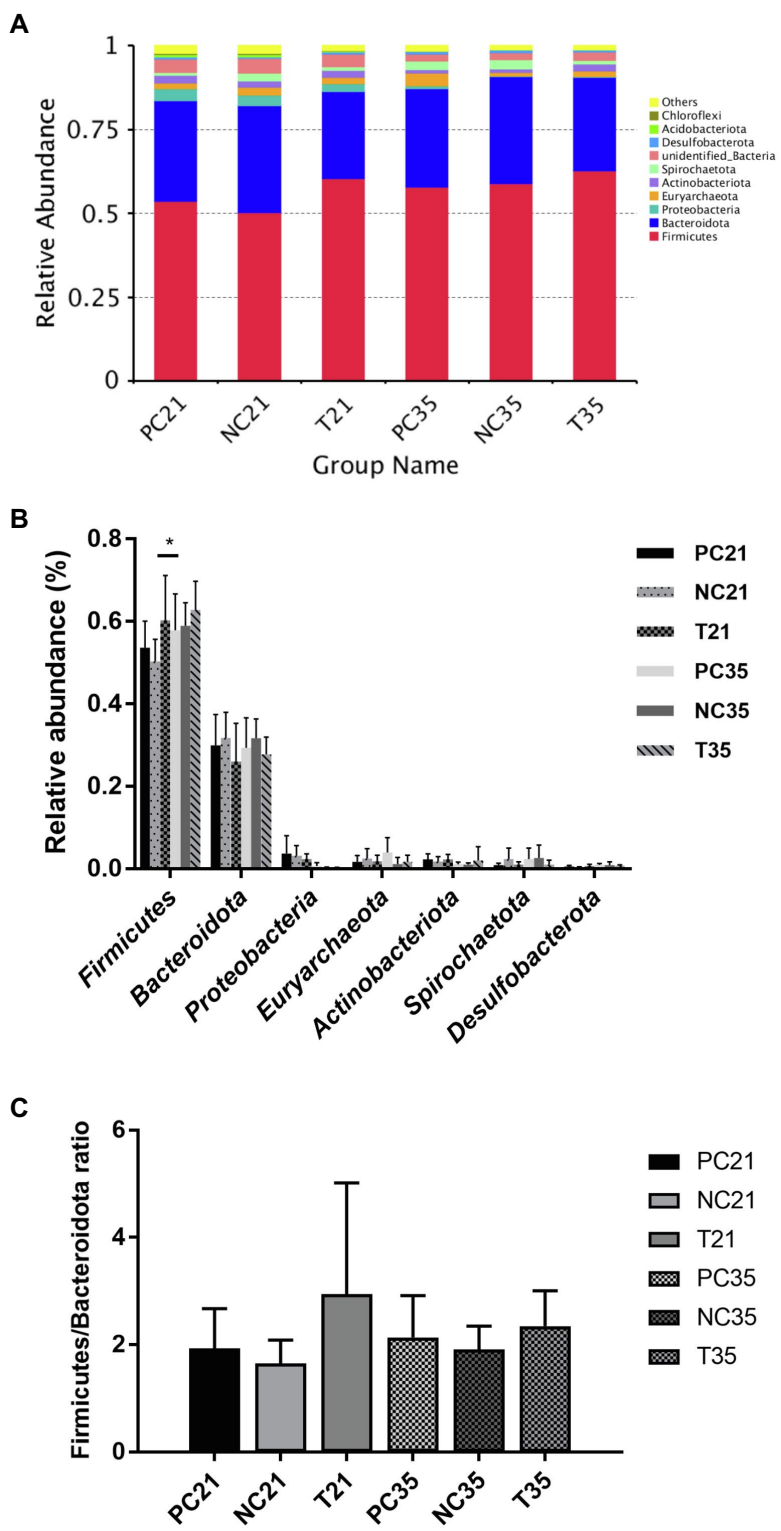
Effects of *Z. Tk* and ZnO on the intestinal microbiota structure at the phylum level in weaned piglets characterized by the taxonomic distributions of the microbial communities in feces at phylum level **(A)**, the top five relatively abundant bacterial phyla detected in fecal samples **(B)**, and the Firmicutes/Bacteroidetes ratio (F/B; **C**). Symbol “*” indicates the significant difference set at the values of *p* of 0.05. Each of the nine samples is selected from each of the three groups of piglets, i.e., the positive control (PC), the negative control (NC), and the test groups, on days 21 and 35, respectively.

The taxa with relative high abundance in the three groups of piglets were analyzed to characterize the changes of the composition in the gut microbiota. The intestinal bacterial composition of each of the three groups of piglets was categorized at the phylum level (Figure 5A). Firmicutes and Bacteroidetes were the two most abundant bacterial phyla in all groups of piglets. Among the top five abundant phyla (Figure 5B), the relative abundance of Firmicutes in the experimental group was significantly increased compared with that of the NC group (*p* < 0.05) in 21 days, while the relative abundance of Actinobacteriota was increased and other three phyla (i.e., Bacteroidota, Proteobacteria, and Euryarchaeota) was decreased in the experimental group compared to those of the NC group (*p* > 0.05). In comparison with the NC group, the relative abundance of three phyla (i.e., Firmicutes, Proteobacteria, and Actinobacteriota) in the PC group showed an increasing trend, while the relative abundance of the other two phyla (i.e., Bacteroidota and Euryarchaeota) showed a decreasing trend. At the age of 35 days, no significant difference was revealed in the bacterial composition among the three groups of intestinal microbiota. However, the relative abundance of Firmicutes and Actinobacteriota in the experimental group increased compared with that of the NC group. Proteobacteria and Euryarchaeota showed increased relative abundance in the PC group compared with the NC group, and the relative abundance of Bacteroidota in the experimental and PC groups decreased in comparison to the NC group. On day 21, the gut microbiota of the experimental group was characterized by an increased Firmicutes/Bacteroidetes ratio (F/B) in comparison to those of the PC and NC groups (*p* < 0.05; Figure 5C). On day 35, the difference in F/B of the three groups was not significantly different.

**Figure 6:**
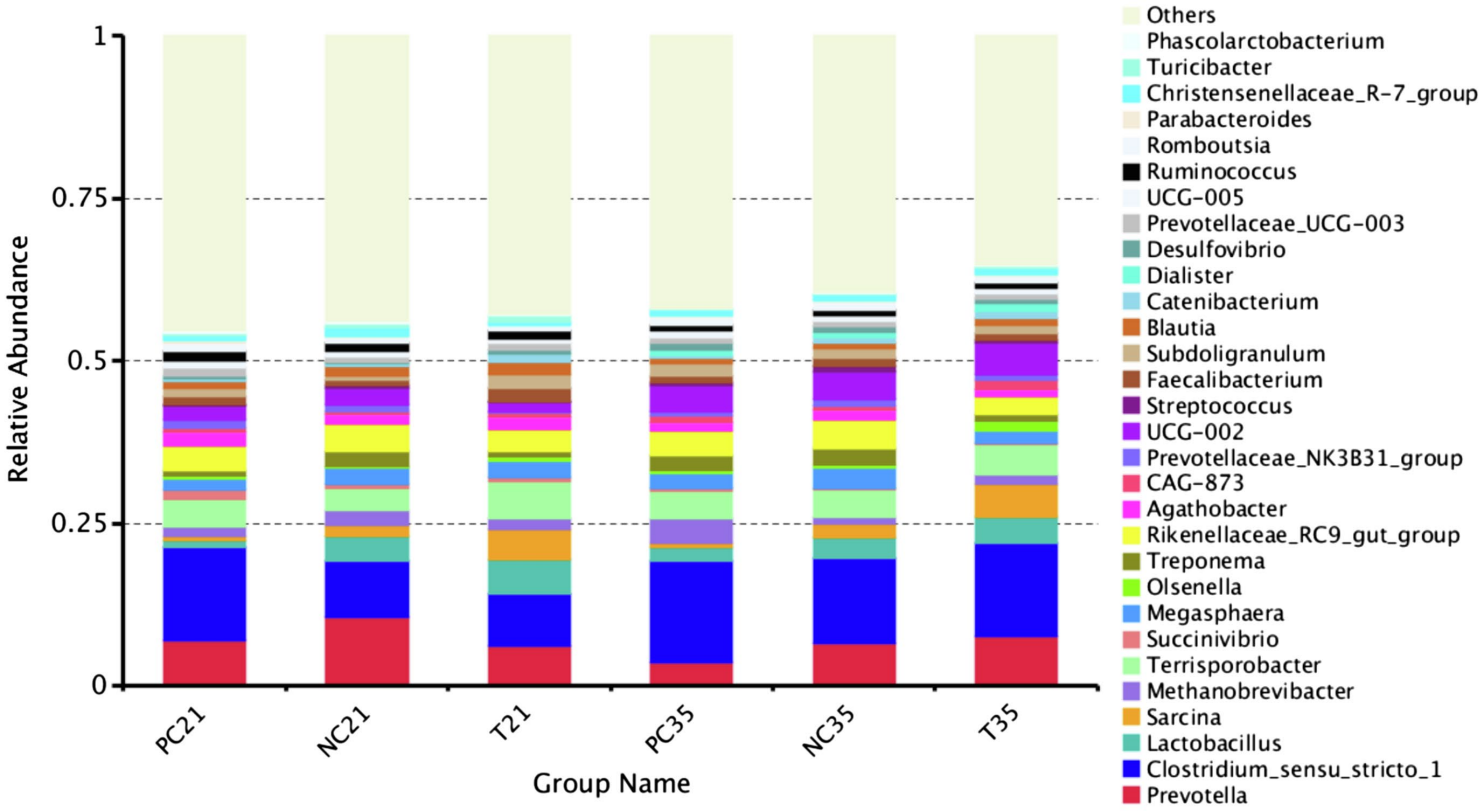
Effects of *Z. Tk* and ZnO on the composition of gut microbiota at the genus level in three groups of piglets on days 21 and 35. Each of the nine samples is selected from each of the three groups of piglets, i.e., the positive control (PC), the negative control (NC), and the test groups, on days 21 and 35, respectively.

The effects of *Z. Tk* on the fecal microbiota in the three groups of weaned piglets were further evaluated based on the top 30 genera with the highest relative abundance (Figure 6). The results showed that at the genus level, the taxonomic structure of the intestinal microbiota of weaned piglets altered with ages and was impacted by both *Z. Tk* and zinc oxide (ZnO). On day 21, the PC group showed increased relative abundance in five genera (i.e., *Clostridium_sensu_stricto_1*, *Terrisporobacter*, *Succinivibrio*, *Olsenella*, and *Agathobacter*) and decreased relative abundance in other six genera (i.e., *Prevotella*, *Lactobacillus*, *Sarcina*, *Methanobrevibacter*, *Megasphaera*, and *Treponema*). In the experimental group, five genera (*Prevotella*, *Clostridium_sensu_stricto_1*, *Methanobrevibacter*, *Treponema*, and *Rikenellaceae_RC9_gut_group*) showed decreased relative abundance, while other five genera (*Lactobacillus*, *Sarcina*, *Terrisporobacter*, *Olsenella*, and *Agathobacter*) showed increased relative abundance compared to that of the NC group. On day 35, the proportions of abundant bacteria in the three groups of piglets altered evidently. For example, three genera (i.e., *Clostridium_sensu_stricto_1*, *Methanobrevibacter*, and *Succinivibrio*) in the PC group showed increased relative abundance, while seven genera (*Prevotella*, *Lactobacillus*, *Sarcina*, *Megasphaera*, *Treponema*, *Rikenellaceae_RC9_obacter group*, and *Agathellaceae_RC9_obacter*) showed decreased relative abundance, compared with that of the NC group. In the test group, seven genera (*Prevotella*, *Clostridium_sensu_stricto_1*, *Lactobacillus*, *Sarcina*, *Methanobrevibacter*, *Terrisporobacter*, and *Olsenella*) and four genera (*Megasphaera*, *Treponema*, *Rikenellaceae_RC9_gut_group*, and *Agathobacter*) showed increased and decreased relative abundance, respectively, in comparison to that of the NC group.

## Discussion

Chinese herbal feed additives have recently gained increasing attention due to their caability of enhancing growth performance by nourshing a healthy gut ecosystem in pigs. Furthermore, the feed additives of plant resources improve both the activity of digestive enzymes in the gastrointestinal tract and the nutrient absorption in pigs (Zhou et al., 2015). Chinese herbal medicines are generally the most economical and labor-effective additives due to their convenient preparation and low cost (Lin et al., 2020). The effect of the fermented herbal additive mixture *Z. Tk* containing *Codonopsis pilosula*, *Radix astragalus*, *R. isatidis*, *R. paeoniae alba*, and *Atractylodes macrocephala* on pig production is unclear. Our study was designed to investigate the effects of *Z. Tk* on the antioxidant capacity, growth performance, and intestinal microbiota of weaned piglets.

Studies have shown that the five herbs used in the mixture in our study contain various types of ingredients with strong antioxidant effects. Specifically, the pectic polysaccharides in *Codonopsis pilosula* could significantly ameliorate the cellular damage caused by H_2_O_2_ treatment and increase the activity of antioxidant enzymes (Zou et al., 2020b). Similarly, the main biologically active components of *Radix astragalus* (e.g., polysaccharides, astragaloside, flavonoids, and saponins) have been revealed to show antioxidant effects (Gong et al., 2018). It has been reported that extracts of *R. astragalus* can decrease the inflammatory response induced by lipopolysaccharide in *Escherichia coli*, while decreasing the release of reactive oxygen species (ROS) and increasing the activation of nuclear factor and the expression of antioxidant cytoprotective factors in cells (Adesso et al., 2018). As the other ingredient in the mixture of *Z. Tk*, *Radix isatidis* is well-known for its broad antiviral activities and antioxidant properties. For example, it was reported that the proteins in *R isatidis* could eliminate the damage of free radicals, showing a strong antioxidant effect *in vivo* (Xiao et al., 2019). Furthermore, ingredients, such as white peony polyphenols, isolated from *R. paeoniae alba* (Zhou et al., 2019) and atractylenolides isolated from *Atractylodes macrocephalae* (Bailly, 2020) are also effective antioxidants. Studies have shown that polysaccharides from *Codonopsis pilosula* can protect the intestinal mucosal immune barrier, potentially maintaining the intestinal microbiota by stimulating the growth of *Lactobacillus* (Fu et al., 2018). The combination of total polysaccharides of *Radix astragalus* and *C. pilosula* was applied to treat 2.5% dextran sulfate sodium induced acute colitis in mice to restore the structure of the intestinal microbiota, increasing the level of *Bacteroidetes* and decreasing the levels of *Firmicutes* and *Proteobacteria* (Tang et al., 2021). The antiviral activities of *R. isatidis* mostly depend on the water-soluble active ingredients, e.g., amino acids, nucleosides, and sulfur-containing alkaloids, to eradicate pathogenic viruses and regulate the immune system (Zhou and Zhang, 2013). Furthermore, the main chemical components of *R. isatidis* (i.e., syringic acid, 2-amino-benzoic acid, and salicylic acid) have shown strong antibacterial activities against the growth of *Escherichia coli*, *Staphylococcus aureus*, and *Shigella dysenteriae* (Kong et al., 2008a,b). Studies have shown that the effective ingredients in *R. paeoniae alba* include paeoniflorin, tannin, and paeonol (Yan et al., 2018). As the most abundant compound reported in *R. paeoniae rubra*, paeoniflorin has been shown strong anti-inflammatory, hepatoprotective, and neuroprotective effects (Yan et al., 2018), displaying an inhibitory effect on biofilm formation of carbapenem-resistant *Klebsiella pneumoniae* (Qian et al., 2020). The sesquiterpenoids, polyacetylenes, and polysaccharides are the most abundant bioactive constituents in *Atractylodes macrocephalae*, which has been traditionally used to treat gastrointestinal hypofunction due to its potential functions of invigorating the spleen and adjusting disordered intestinal microbiota (Zhu et al., 2018). In rat models with disrupted intestinal microbiota, an administration of polysaccharides in *A. macrocephalae* significantly improved the rat vigor and body weight, promoting the ability of intestinal bacteria to digest sugars (Wang et al., 2014). These results suggest that the biologically active ingredients of the five traditional Chinese medicines mixed in *Z. Tk* are of significant importance for adjusting the function of gastrointestinal tract, affecting the structure of the intestinal microbiota, and reducing diarrhea caused by weaning stress in the weaned piglets.

### Antioxidant Effect of *Z. Tk*

Weaning stress causes several health problems (e.g., diarrhea, growth restriction, and intestinal dysfunction) in piglets, as the animals are rapidly forced to adjust to the nutritional, immunological, and psychological changes (Cao et al., 2018). Furthermore, weaning stress disrupts free-radical metabolism and antioxidative system, causing severe oxidative stress in animals (Yin et al., 2014; Cao et al., 2018). The intestines are the main digestive and absorptive organ for nutrients, providing a selective blockage preventing various types of antigens. It has been reported that a healthy intestinal environment plays an essentially important role in maintaining an organism’s healthy condition (Thoo et al., 2019; Wan et al., 2019), while the oxidative stress is generally considered as a critical factor involved in the disruption of a healthy intestinal ecosystem. Therefore, it is practically important to identify appropriate natural feed additives as therapeutic agents to lessen the diseases related to oxidative stress (Zhuang et al., 2019).

Our results showed that the treatment of *Z. Tk* with appropriate concentrations and treatment time significantly increased the IPEC-J2 cell viability. H_2_O_2_ significantly downregulated the expression of GSH-Px, while *Z. Tk* significantly improved the T-AOC and the expression of GSH-Px in IPEC-J2 cells. These results are consistent with those reported previously, suggesting that the maintenance of strong antioxidant activities in weaned piglets is beneficial to support the intestinal barrier function (Chen et al., 2018a). Furthermore, a healthy intestinal environment relies on the tight junction proteins, which are the major factors determining the intestinal barrier function by sealing the paracellular space among epithelial cells (Nunes et al., 2019). In our study, the expression of three genes (i.e., *occludin*, *claudin*, and *ZO-1*) encoding three major intestinal barrier proteins (Liao et al., 2017) was significantly decreased by the treatment of H_2_O_2_ in the IPEC-J2 cells. These similar results were also reported previously (Cao et al., 2020). The *Z. Tk* significantly attenuated the dysfunction of the intestinal barrier by upregulating the expression of genes encoding the tight junction proteins under H_2_O_2_-induced oxidative stress in the IPEC-J2 cells. In short, the *Z. Tk* significantly decreased the H_2_O_2_-induced cell damage by increasing the cell viability and improving the antioxidant capacity in IPEC-J2 cells. It was reported that two types of polysaccharides isolated from *Codonopsis pilosula* (one of the five species of Chinese medicinal herbs used to make the mixture of *Z. Tk*) showed antioxidant ability against intestinal epithelial cells and proved to be effective prebiotics (Zou et al., 2020c). These results demonstrated that *Z. Tk* has shown the potential to attenuate intestinal injury (i.e., the intestinal barrier disruption) mainly by upregulating the oxidative status in the cells.

### Effects of *Z. Tk* on Growth Performance in Piglets

In the swine industry, the feed efficiency is very important for the growth and fattening of animals. It is desired to generate a large amount of meat with less consumption of feed. We investigated the effect of *Z. Tk* on the growth performance of weaned piglets was investigagted with the goal to use it as a substitute for ZnO as a feed additive for weaned piglets. In our study, the addition of ZnO (*p* < 0.01) and *Z. Tk* (*p* < 0.05) to the feed both significantly improved the FCR of weaned piglets during the 1–35 days of post-weaning period, which effectively improved the economic benefits. Furthermore, the addition of *Z. Tk* to the feed significantly increased the BW at 21 and 35 days (*p* < 0.05). Our findings showed that the dietary supplementation of *Z. Tk* significantly improved the growth performance of early weaned piglets as demonstrated by several indicators, including the BW, ADG, ADFI, FCR, and diarrhea rate, suggesting that *Z. Tk* effectively helps piglets overcome the weaning stress at the early production stages. The similar results were reported previously, suggesting a positive effect of a diet supplemented with a mixture of Chinese herbal medicines on the growth performance of pigs (Yeh et al., 2011; Abdallah et al., 2019). Lan et al. (2017) reported that the dietary supplementation of a mixture containing *Astragalus*, *Codonopsis*, and allicin increased the growth performance, the nutrient digestibility, the intestinal microbial balance (i.e., increased amount of *Lactobacillus* and decreased amount of *E. coli*), the immune response, and the meat quality of pigs.

The application of microbial fermentation to Chinese herbal medicines largely upgrades the functions of microorganisms and Chinese herbal medicines, showing enhanced effects when used in combination than applied alone. The active ingredients in the Chinese herbal medicines are released in the presence of enzymes provided by the microorganisms, effectively improving the efficacy of the herbal medicines (Ai et al., 2019; Li et al., 2020). Furthermore, after the microbial fermentation, the active macromolecular substances in the herbal medicines are transformed into small molecules that can be directly absorbed in the animal’s intestines and fully metabolized in the body. Therefore, the drug residues are partly avoided (Ai et al., 2019; Li et al., 2020). The prebiotics produced by the herbal medicines after fermentation promote the reproduction of microorganisms, while the microorganisms improve the absorption of the herbal medicines in the body, complementing each other and ultimately enhancing the therapeutic effect (Ai et al., 2019). Furthermore, the process of inactivation of probiotics and degradation of metabolites after fermentation may affect the active ingredients in the herbal medicines, which is unfavorable in practice and should be avoided.

### Effects of *Z. Tk* on the Microbial Diversity of the Intestinal Microbiota in Piglets

The understanding of the relationship among gut microbiota and growth performance will help us to effectively enhance the porcine growth and fattening performance of pigs. The bacterial communities of the fecal samples were compared among the NC, PC, and experimental groups of piglets on days 21 and 35 in our study. Results showed that no significant difference of the alpha diversities was revealed between the three groups of animals (Figure 3), suggesting that species richness and diversity of the bacterial communities were not affected significantly by either ZnO or *Z. Tk* during the entire experiment of this study. However, as the animals aged, the alpha diversity indices (i.e., Chao1, Shannon, and observed-species) of weaned piglets decreased. Studies have reported various types of changing patterns in alpha diversity indices. For example, the alpha diversities of gut microbiota increased with aging in pigs (Upadrasta et al., 2013; Niu et al., 2015), while Frese et al. (2015) reported that the alpha diversities initially increased gradually from birth to 21 days and then plateaued from 21 to 42 days. Furthermore, Arfken et al. (2020) reported that the microbial diversity and species richness and evenness were stable or showed slight decline 24 days after birth. These results suggested that the increase in the diversity and richness of the intestinal flora of piglets mainly occurs before the 21st day of birth and tends to stabilize or decrease slightly after weaning. In our study, the microbial alpha index of piglets at 35 days after weaning (56 days of age) decreased compared with that of 21 days after weaning (42 days of age). Results of beta diversity analysis showed evidently the differences between the NC and experimental groups based on the PCoA. For example, the NC and PC groups were closely related to each other on day 21, while the three groups of piglets were clustered together on day 35, indicating that the influential effects of *Z. Tk* on the taxonomic structure of the intestinal microbiota on day 21 gradually became less evident as the age increased to 35 days.

### Effects of *Z. Tk* on Taxonomic Composition of the Intestinal Microbiota in Piglets

Our results revealed that all samples were relatively dominated by Firmicutes, Bacteroidota, and Proteobacteria at the phylum level, similar to the results previously reported (Han et al., 2017), and were dominated by *Prevotella*, *Clostridium_sensu_stricto_1*, and *Lactobacillus* at the genus level. Studies have shown that the taxa of Firmicutes and Bacteroidetes dominate the human, mouse, and pig microbiota (Ley et al., 2005), while lean and obese pigs contain different proportions of Firmicutes and Bacteroidetes (Guo et al., 2008). Specifically, the levels of Bacteroidetes and Bacteroides were lower in obese pigs than those of the lean pigs, while significant difference of the level of Firmicutes was not revealed between obese and lean pigs. Furthermore, the levels of Bacteroidetes and Firmicutes in the ceca also differ between obese and lean mice with the genetically obese mice showing 50% decrease in the abundance of Bacteroidetes and a proportional increase in the level of Firmicutes, in comparison to lean mice (Ley et al., 2005). It was reported that the microbiota of obese mice enhanced energy generation from ingested diet (Turnbaugh et al., 2006), while the overweight and obese subjects presented low level of Bacteroidetes (Ley et al., 2006). Generally considered as a biomarker to evaluate the body weight gain performance (Ding et al., 2019), the F/B is correlated with body mass index and tends to increase in stout healthy subjects compared to the lean healthy subjects (Ding et al., 2019). Similar results were revealed in our study. Specifically, the F/B of the experimental group increased significantly in animals with larger BW in 21 days of weaning. In our study, the weaned piglets supplemented with *Z. Tk* in the diet were characterized by the increased level of Firmicutes and decreased level of Bacteroides. These results were consistent with the significant increase in body weight of the weaned piglets, suggesting that the mixture of *Z. Tk* improved the weight of piglets by altering the bacterial structure of the intestinal microbiota (i.e., *Firmicutes* and *Bacteroides*).

Several studies on animals during their weaning stages have shown a decreased relative abundance of the *Lactobacillus* group, whereas the bacteria in *Clostridium*, *Prevotella*, and facultative anaerobes, such as *Proteobacteriaceae* (e.g., *E. coli*) were generally positively impacted (Gresse et al., 2017). Weaned piglets are generally supplemented with beneficial bacteria to counteract the disturbance of the intestinal microbiota. In particular, species of *Lactobacillus* are major players in disease prevention, while the rapid decrease in their relative abundance in the gut microbiota during weaning transition contributes to an increased risk of diseases (Konstantinov et al., 2006). In our study, the relative abundance of *Lactobacillus* in the experimental group of piglets fed with *Z. Tk* showed an increasing trend from days 21 to 35, indicating that the supplementation of fermented herbal mixture is beneficial to the healthy development of the intestinal microbiota of piglets after weaning. Furthermore, the pathogenic bacteria decreased in the experimental group (Figure 6). For example, the relative abundance of *Proteobacteria* in the experimental group showed a decreasing state in 21 days. Studies have shown that *Proteobacteria* is significantly increased in the intestines of mice with ulcerative colitis producing a large number of pro-inflammatory cytokines (Powell et al., 2012). These results suggest that the chronic enrichment of members of *Proteobacteria* in the intestine may represent either an unbalanced and unstable microbial community structure or the unhealthy state of the host, while the supplementation of *Z. Tk* reduces the risk of intestinal diseases. In our study, *Prevotella* responded differently to the treatments of ZnO and *Z. Tk*. Specifically, the relative abundance of *Prevotella* decreased in both periods of 21 days and 35 days under the treatment of ZnO, whereas decreased in 21 days and then increased in 35 days under the treatment of *Z. Tk*. *Prevotella* contains a group of Gram-Negative and obligate anaerobic bacilli with the capability of fermenting carbohydrates to generate short chain fatty acids including acetate and butyrate, exerting an anti-inflammatory effect on immune cells and ultimately inhibiting the growth of potentially pathogenic bacteria (Rivera-Chávez et al., 2016; Zou et al., 2020a). In the PC group, the relative abundance of *Clostridium_sensu_stricto_1* and *Sarcina* showed an increasing and decreasing trends, respectively, in both periods of 21 and 35 days. However, in the experimental group, the relative abundance of *Clostridium_sensu_stricto_1* was not significantly different on either 21 or 35 days, while *Sarcina* showed an upward trend. Studies have shown that the *Clostridium_sensu_stricto_1* contains harmful bacteria with adverse effects on the intestinal tract (Lin et al., 2018; Zou et al., 2020a). Species in *Sarcina* are anaerobic Gram-positive cocci and associated with delayed gastric emptying (Lam-Himlin et al., 2011), while these microorganisms do not appear to cause direct mucosal injury but always appear in the gastrointestinal tract enrichment in the intestines of patients (Chan et al., 2020). Our results indicate that the treatment of *Z. Tk* and ZnO did not show any inhibitory effect on some Gram-positive pathogenic bacteria, such as *Clostridium_sensu_stricto_1* and *Sarcina*, while *Z. Tk* showed inhibitory effect on gram-negative pathogenic bacteria, such as Proteobacteria, simultaneously, promoting the relative abundance of beneficial bacteria, such as *Lactobacillus* and ultimately helping weaned piglets to smoothly survive the weaning stress.

## Conclusion

In this study, we have demonstrated that the fermented Chinese herbal mixture *Z. Tk* can not only improve the intestinal antioxidant capacity and repair the intestinal cell barrier *in vitro* but also enhance the growth performance and the taxonomic structure of the intestinal microbiota of the weaned piglets. The intestinal microbiota of weaned piglets was altered by the addition of *Z. Tk*, mainly increasing the relative abundance of beneficial bacteria, such as *Lactobacillus* and inhibiting Gram-negative pathogenic bacteria such as members of *Proteobacteria*. More importantly, these bacteria helped to maintain the intestinal microbiota at a favorable state for the host, effectively reduced the abnormal changes in the intestinal structure of microbiota in weaned piglets and created a favorable microbial community for the subsequent restoration of a balanced gut microbiota. Our study demonstrates that *Z. Tk* is potentially a promising substitute of ZnO for facilitating the weaning phase and improving production efficiency of piglets. Specifically, *Z. Tk* showed significantly improved the growth performance of weaned piglets in comparison to that of ZnO additives. We note that because the weaned piglets have not been slaughtered due to the experimental conditions in this study, more studies *in vivo* are needed to verify the antioxidant capacity of *Z. Tk* revealed in this study.

## Data Availability Statement

The datasets presented in this study can be found in online repositories. The names of the repository/repositories and accession number(s) can be found at: https://www.ncbi.nlm.nih.gov/, PRJNA714150.

## Ethics Statement

The animal study was reviewed and approved by Committee on the Ethics of Animal Experiments of the College of Veterinary Medicine, Huazhong Agricultural University.

## Author Contributions

YX and YL: conceptualization. YW, WL, ST, XY, and JL: methodology. YL and TS: resources. YH: data curation and writing – original draft preparation. YH and YX: writing – review and editing. YX: supervision and funding acquisition. YL, TS, ZZ, and YX: project administration. All authors contributed to the article and approved the submitted version.

## Funding

This research was funded by the Innovative Job Funds of Agricultural Science and Technology of Hubei Province, grant number 2019-620-000-001-30.

## Conflict of Interest

YL, WL, and ST are employed by COFCO Feed Co., Ltd. TQ and XL are employed by Hubei Huada Real Science & Technology Co., Ltd.The remaining authors declare that the research was conducted in the absence of any commercial or financial relationships that could be construed as a potential conflict of interest.

## Publisher’s Note

All claims expressed in this article are solely those of the authors and do not necessarily represent those of their affiliated organizations, or those of the publisher, the editors and the reviewers. Any product that may be evaluated in this article, or claim that may be made by its manufacturer, is not guaranteed or endorsed by the publisher.

## Supplementary Material

Supplementary Table S1 Feed formula of different groups of piglets.

Supplementary Table S2 Formula nutrient values.

Supplementary Table S3 Summary of sequencing data of each sample.

Supplementary Figure S1 The rarefaction curve.

## Abbreviations

*Z. Tk*: Fermented Chinese herbal mixture *Zhihuasi Tk*
ZnO: Zinc oxide
TCM: Traditional Chinese medicine
IPEC-J2: The porcine small intestinal epithelial cell line
GSH-Px: Glutathione peroxidase
T-AOC: Total antioxidant capacity
BW: Body weight
ADG: Average daily gain
ADFI: Average daily feed intake
FCR: Feed conversion rate
F/B: Ratio of Firmicutes/Bacteroidetes
