# Supplementary material for: Mixture of Five Fermented Herbs (Zhihuasi Tk) Alters the Intestinal Microbiota and Promotes the Growth Performance in Piglets
Source: Front Microbiol. 2021 Oct 26;12:725196. doi: 10.3389/fmicb.2021.725196 (PMC8576326; doi:10.3389/fmicb.2021.725196)
Supplement: Supplementary Table S1 — Feed formula of different groups of piglets. [file Data_Sheet_1.docx]

Supplementary Material

# Supplementary Data

None

# Supplementary Figures and Tables

## Supplementary Figures


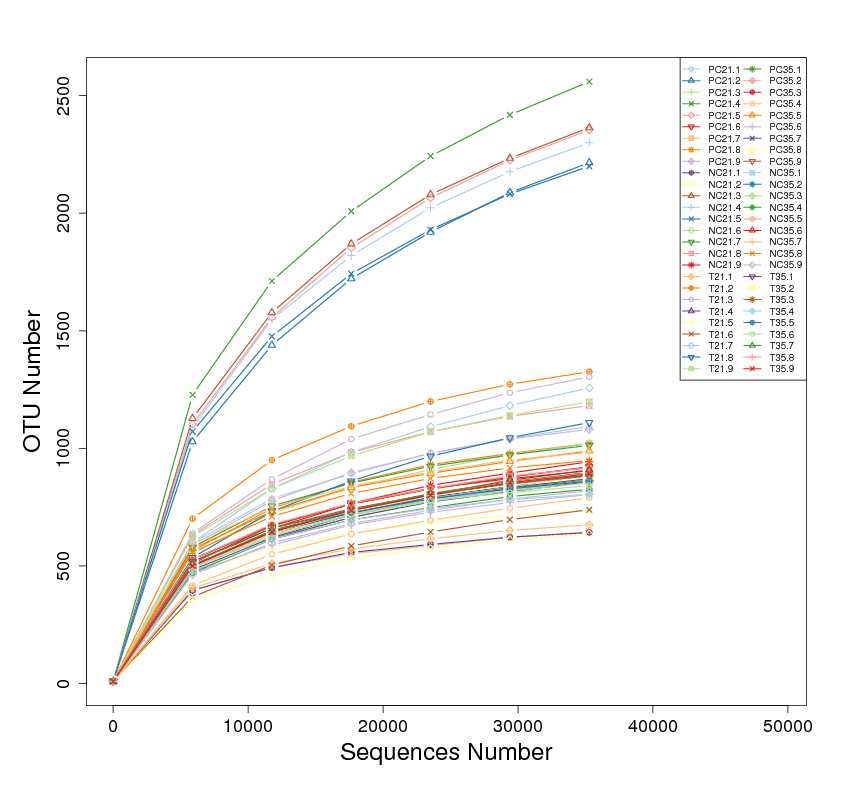


**Supplementary Figure 1.** The rarefaction curve

## Supplementary Tables

# Supplementary Table 1. Feed formula of different treatment groups (%)

|  | NC (basal diet) | PC | T |
| --- | --- | --- | --- |
| Maize (for dairy breeding pigs) | 43.19 | 43.19 | 43.19 |
| Puffed corn | 17.00 | 17.00 | 17.00 |
| Soybean meal 46 | 14.00 | 14.00 | 14.00 |
| Soybean oil (grade IV) | 1.08 | 1.08 | 1.08 |
| Whey powder | 7.50 | 7.50 | 7.50 |
| Fermented soybean meal | 7.50 | 7.50 | 7.50 |
| Super steam fishmeal (CP65%) | 4.00 | 4.00 | 4.00 |
| CaHPO4 | 0.74 | 0.74 | 0.74 |
| D-Glucose monohydrate | 1.20 | 1.20 | 1.20 |
| fine ground limestone | 0.47 | 0.47 | 0.47 |
| L-Lysinesulphate（70%） | 0.89 | 0.89 | 0.89 |
| Acidulant | 0.50 | 0.50 | 0.50 |
| DL-2-Amino-4-(methylthio)butyric acid; | 0.28 | 0.28 | 0.28 |
| L-Threonine | 0.24 | 0.24 | 0.24 |
| NaCl | 0.20 | 0.20 | 0.20 |
| trace mineral feed | 0.15 | 0.15 | 0.15 |
| Valine | 0.12 | 0.12 | 0.12 |
| Tryptophan | 0.09 | 0.09 | 0.09 |
| Choline chloride 60% | 0.08 | 0.08 | 0.08 |
| vitamine premix | 0.04 | 0.04 | 0.04 |
| Antioxidants | 0.02 | 0.02 | 0.02 |
| Complex enzyme | 0.02 | 0.02 | 0.02 |
| Phytase (10,000 units) | 0.01 | 0.01 | 0.01 |
| ZnO |  | 0.20 |  |
| Z.TK |  |  | 0.40 |
| maifanstone | 0.68 | 0.47 | 0.17 |
| Total | 100.00 | 100.00 | 100.00 |

# Supplementary Table 2. Formula nutrient value

| index | Nutrient value（%） |
| --- | --- |
| H_2_O | 12.00 |
| Crude protein | 19.50 |
| Crude fiber | 3.10 |
| Crude fat | 4.76 |
| Crude Ash | 5.20 |
| Lys | 1.46 |
| Met | 0.57 |
| Thr | 0.96 |
| Trp | 0.30 |
| Val | 1.01 |
| SID Lys | 1.30 |
| SID Met | 0.52 |
| SID Thr | 0.82 |
| SID Trp | 0.26 |
| SID Val | 0.86 |
| Ca | 0.68 |
| P | 0.64 |
| STTD P | 0.37 |
| DE（kcal/kg） | 3400 |
| NE（kcal/kg） | 2330 |

# Supplementary Table 3. Summary of sequencing data for each sample

| Sample Name | Raw PE(#) | Raw Tags(#) | Clean Tags(#) | Effective Tags(#) | Base(nt) | AvgLen(nt) | Q20 | Q30 | GC% | Effective% |
| --- | --- | --- | --- | --- | --- | --- | --- | --- | --- | --- |
| T21.1 | 91,744 | 89,647 | 87,571 | 59,743 | 24,668,844 | 413 | 98.5 | 95.08 | 53.59 | 65.12 |
| T21.2 | 82,274 | 77,916 | 75,040 | 49,474 | 20,468,344 | 414 | 97.9 | 93.47 | 54.18 | 60.13 |
| T21.3 | 93,721 | 90,272 | 87,296 | 58,687 | 24,465,319 | 417 | 98.13 | 94.2 | 54.15 | 62.62 |
| T21.4 | 82,181 | 78,374 | 75,494 | 51,417 | 21,300,673 | 414 | 98.01 | 93.71 | 53.97 | 62.57 |
| T21.5 | 81,054 | 78,992 | 77,100 | 54,561 | 22,574,903 | 414 | 98.34 | 94.71 | 54.25 | 67.31 |
| T21.6 | 88,141 | 86,379 | 84,652 | 60,302 | 24,904,440 | 413 | 98.42 | 94.88 | 53.4 | 68.42 |
| T21.7 | 99,314 | 96,642 | 94,442 | 66,024 | 27,207,984 | 412 | 98.35 | 94.75 | 53.32 | 66.48 |
| T21.8 | 97,773 | 95,208 | 92,699 | 64,966 | 26,960,547 | 415 | 98.29 | 94.53 | 54.63 | 66.45 |
| T21.9 | 80,792 | 78,407 | 76,482 | 52,273 | 21,561,406 | 412 | 98.29 | 94.54 | 53.71 | 64.7 |
| T35.1 | 90,896 | 89,604 | 86,004 | 53,777 | 22,291,277 | 415 | 98.53 | 95.15 | 53.52 | 59.16 |
| T35.2 | 84,115 | 82,933 | 80,176 | 55,093 | 22,767,300 | 413 | 98.64 | 95.42 | 53.88 | 65.5 |
| T35.3 | 85,737 | 84,376 | 80,362 | 54,725 | 22,651,015 | 414 | 98.48 | 95.03 | 53.94 | 63.83 |
| T35.4 | 80,585 | 79,272 | 76,229 | 50,027 | 20,583,015 | 411 | 98.46 | 94.87 | 53.56 | 62.08 |
| T35.5 | 87,223 | 86,053 | 83,072 | 52,829 | 21,780,259 | 412 | 98.63 | 95.35 | 53.45 | 60.57 |
| T35.6 | 73,348 | 72,237 | 68,856 | 46,332 | 19,149,913 | 413 | 98.46 | 94.92 | 53.99 | 63.17 |
| T35.7 | 90,556 | 89,292 | 86,001 | 58,727 | 24,200,220 | 412 | 98.53 | 95.08 | 53.35 | 64.85 |
| T35.8 | 99,863 | 98,322 | 94,838 | 61,452 | 25,326,331 | 412 | 98.62 | 95.4 | 55.16 | 61.54 |
| T35.9 | 92,835 | 91,634 | 88,597 | 61,462 | 25,432,414 | 414 | 98.69 | 95.5 | 53.29 | 66.21 |
| PC21.1 | 85,829 | 85,116 | 83,658 | 60,008 | 25,068,336 | 418 | 98.89 | 96.17 | 54.19 | 69.92 |
| PC21.2 | 79,177 | 77,274 | 75,296 | 50,408 | 20,958,425 | 416 | 98.32 | 94.59 | 53.26 | 63.66 |
| PC21.3 | 84,847 | 83,058 | 80,632 | 54,253 | 22,477,742 | 414 | 98.13 | 94.05 | 53.04 | 63.94 |
| PC21.4 | 84,432 | 83,012 | 81,398 | 56,240 | 23,250,451 | 413 | 98.52 | 95.11 | 54.02 | 66.61 |
| PC21.5 | 90,096 | 87,498 | 85,000 | 57,498 | 23,815,096 | 414 | 98.31 | 94.66 | 53.82 | 63.82 |
| PC21.6 | 86,479 | 84,330 | 82,322 | 56,154 | 23,123,815 | 412 | 98.36 | 94.72 | 54.53 | 64.93 |
| PC21.7 | 84,675 | 82,453 | 80,491 | 59,048 | 24,363,604 | 413 | 98.44 | 94.97 | 54.41 | 69.73 |
| PC21.8 | 87,307 | 86,202 | 84,225 | 62,723 | 25,920,357 | 413 | 98.74 | 95.88 | 54.02 | 71.84 |
| PC21.9 | 81,760 | 80,973 | 79,872 | 60,813 | 25,099,263 | 413 | 99.04 | 96.57 | 54.45 | 74.38 |
| PC35.1 | 94,135 | 91,952 | 89,033 | 57,579 | 23,636,892 | 411 | 98.65 | 95.45 | 53.67 | 61.17 |
| PC35.2 | 80,526 | 79,330 | 76,268 | 51,429 | 21,241,907 | 413 | 98.66 | 95.48 | 53.52 | 63.87 |
| PC35.3 | 98,142 | 96,659 | 93,301 | 59,981 | 24,635,846 | 411 | 98.62 | 95.32 | 53.31 | 61.12 |
| PC35.4 | 74,937 | 73,905 | 71,405 | 46,278 | 19,080,861 | 412 | 98.65 | 95.45 | 53.55 | 61.76 |
| PC35.5 | 89,676 | 87,960 | 85,387 | 57,237 | 23,691,252 | 414 | 98.7 | 95.57 | 54.23 | 63.83 |
| PC35.6 | 91,877 | 90,548 | 87,141 | 57,634 | 23,796,576 | 413 | 98.53 | 95.15 | 54.14 | 62.73 |
| PC35.7 | 91,849 | 90,480 | 87,412 | 58,692 | 24,201,429 | 412 | 98.66 | 95.45 | 53.69 | 63.9 |
| PC35.8 | 89,929 | 88,790 | 85,745 | 57,768 | 23,952,562 | 415 | 98.67 | 95.49 | 53.94 | 64.24 |
| PC35.9 | 98,396 | 96,806 | 93,606 | 59,365 | 24,471,747 | 412 | 98.63 | 95.36 | 54 | 60.33 |
| NC21.1 | 94,268 | 92,961 | 91,326 | 60,149 | 24,822,767 | 413 | 99 | 96.42 | 53.49 | 63.81 |
| NC21.2 | 92,878 | 91,370 | 90,087 | 60,678 | 25,094,675 | 414 | 99.11 | 96.74 | 54.58 | 65.33 |
| NC21.3 | 88,519 | 86,597 | 84,579 | 57,277 | 23,642,447 | 413 | 98.36 | 94.67 | 54.35 | 64.71 |
| NC21.4 | 99,627 | 98,125 | 96,738 | 65,850 | 27,148,035 | 412 | 99.08 | 96.65 | 54.3 | 66.1 |
| NC21.5 | 84,364 | 83,367 | 82,245 | 60,125 | 24,769,589 | 412 | 99.13 | 96.81 | 54.45 | 71.27 |
| NC21.6 | 93,275 | 91,428 | 89,339 | 65,214 | 27,077,517 | 415 | 98.38 | 94.79 | 54.33 | 69.92 |
| NC21.7 | 88,339 | 86,826 | 85,268 | 60,000 | 24,744,556 | 412 | 98.63 | 95.4 | 53.72 | 67.92 |
| NC21.8 | 71,041 | 67,915 | 65,548 | 44,355 | 18,371,626 | 414 | 97.96 | 93.63 | 53.99 | 62.44 |
| NC21.9 | 77,324 | 74,786 | 72,513 | 46,606 | 19,288,003 | 414 | 98.29 | 94.59 | 54.05 | 60.27 |
| NC35.1 | 93,599 | 92,213 | 89,208 | 59,988 | 24,654,276 | 411 | 98.55 | 95.16 | 53.92 | 64.09 |
| NC35.2 | 81,652 | 80,347 | 77,599 | 50,556 | 20,837,252 | 412 | 98.52 | 95.03 | 53.92 | 61.92 |
| NC35.3 | 82,150 | 81,063 | 78,457 | 50,411 | 20,741,281 | 411 | 98.72 | 95.6 | 53.66 | 61.36 |
| NC35.4 | 64,746 | 59,345 | 57,146 | 37,062 | 15,270,341 | 412 | 98.12 | 94.11 | 53.92 | 57.24 |
| NC35.5 | 92,815 | 91,445 | 88,387 | 55,694 | 22,987,588 | 413 | 98.59 | 95.23 | 53.69 | 60.01 |
| NC35.6 | 94,488 | 93,162 | 88,833 | 57,486 | 23,779,518 | 414 | 98.53 | 95.17 | 54.66 | 60.84 |
| NC35.7 | 93,592 | 92,503 | 89,454 | 57,969 | 24,000,543 | 414 | 98.68 | 95.57 | 54.51 | 61.94 |
| NC35.8 | 83,055 | 76,617 | 73,297 | 48,451 | 20,100,035 | 415 | 98.23 | 94.39 | 54.57 | 58.34 |
| NC35.9 | 92,628 | 91,512 | 88,432 | 57,921 | 23,973,255 | 414 | 98.66 | 95.49 | 54.19 | 62.53 |
